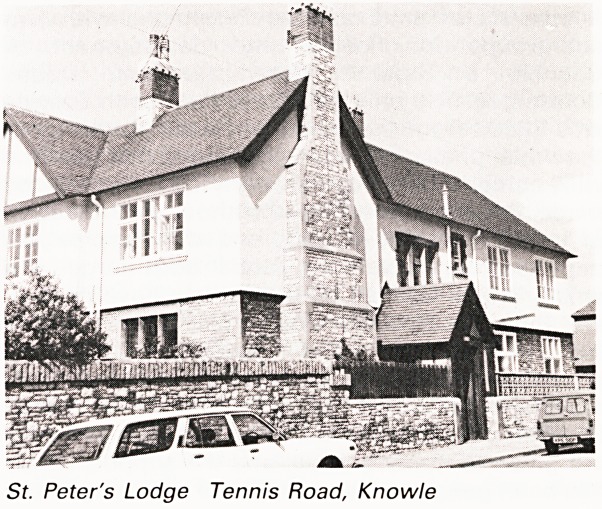# St. Peter's Hospice, the Evolution of Terminal Care for Cancer Patients in Bristol

**Published:** 1981

**Authors:** H. K. Bourns

**Affiliations:** Consultant Surgeon, Bristol Royal Infirmary


### St. Peter's Hospice The Evolution of Terminal Care for Cancer Patients in Bristol

**H. K. Bourns**
Consultant Surgeon, Bristol Royal Infirmary

Malignant tumours, although with modern methods often curable, are still one of the most frequent causes of death.

The control of terminal symptoms such as pain, vomiting and anxiety requires very special skills not always readily available within a large Teaching Hospital or a District Hospital, both of which are, of necessity, mainly orientated towards curative treatment.

The Department of Health and Social Security in Health Trends (Vol.10, 1978) states that 'Given the support of an interested general practitioner, preferably backed up by a domiciliary care service based on a hospice, many patients can receive all the necessary care in their own homes where the practitioner will have the responsibility for co-ordinating treatment. This is usually preferred by the patient and his relatives providing that symptoms are adequately controlled and there is assurance of immediate admission to a hospice if this proves medically or socially necessary'.

A Working Group for Terminal Care was set up in March 1979 with the following Terms of Reference –

'To consider the organisation of primary, continuing and terminal care services for cancer and to make recommendations' issued a Report in March 1980. One of the conclusions is 'that every Regional Health Authority should plan for terminal care provision taking into account both NHS and voluntary resources and after consultation with these voluntary bodies which fund terminal care services.

In Bristol a group of Doctors and lay people met between May and June 1969 with the following Terms of Reference – 'To consider the needs for accommodation and care of terminal illness in Bristol and environs and to report'. A Report was published in August 1969. The Chairman of the group was the late Professor A. V. Neale. His sudden and untimely death left the group in a dejected and unhappy state. In 1973 efforts were made to restart it after a meeting in the Victoria Rooms attended by a large number of interested medical and lay people. Mr. A. M. Urquhart took over the Chairmanship of a small group in 1974 and after visiting many buildings either vacant or about to fall vacant, none of which were either suitable or economical, a purpose-built unit in the grounds of Southmead Hospital was planned. The cost made this project impossible, so St. Peter's Hospice was conceived as a Domiciliary Service which could be started without any building. We received a donation of £5000 and an interest-free loan of a similar sum in 1976. The first nurse was appointed in January 1977 and started work based on office accommodation lent to St. Peter's Hospice by Bristol District in Lawrence Hill Health Centre.

*St. Peter's Lodge*
*Headquarters of the Home-Care Service and tranquil setting for in-patient care.*

The Home Care team gradually increased and a unit of 4 nurses was created and served an area within a 6 mile radius round the Health Centre. St. Peter 's Nurses visited patients referred by the local General Practitioners, and worked very closely with the District Nurses and Health Visitors. The Nurses had many meetings with the District Nurses of the Bristol Districts, and from the outset it was made clear that they were 'sharing the caring' with both the relatives and the National Health Service personnel.

It soon became clear that St. Peter's Hospice needed some beds for care of patients either when home circumstances were being over-taxed and likely to break down, or when some symptoms were becoming impossible to control on a domiciliary basis. A Victorian house in Tennis Road, Knowle, owned by the Sisters of Charity became vacant, and the Trustees offered it to St. Peter's after terms had been agreed. A body of volunteers under the direction of skilled builders and decorators quickly transformed the Lodge so that the first patients could be admitted in May 1979.

The Domiciliary Service office moved to St. Peter's Lodge, and the local unit consisted of a Medical Director, a General Practitioner and a Radiotherapist, and for the first time the 'Home Care Team' could admit patients to beds within their own organisation. Patients are often admitted more than once during the course of the terminal illness, and in a recent survey it was noted that the average time spent in the Hospice by the patients was about 21 days. Roughly one third of the patients die in the home, and two thirds are discharged to the care of the relatives backed up by the Domiciliary Nurses.

During 1980 it became obvious that the Service should be expanded to cover all Bristol and its surrounding areas, so a second team of nurses was engaged and based on Clifton – thus the two teams share both North and South Bristol.

The Home Care Nurses have special knowledge and have worked either in other hospices or have been sent on courses in other units, and so they bring an expertise to their work which is appreciated by both patients and local health agents alike. The Hospice service cannot stop the progress of the disease, but there is always something that can be done. The aim is not so much to help people to die as to help them to live until they do die. In Dr. Lamaton's words 'to make the body a comfortable place to live in'.

**TIME**

Nurses can spend many hours on a visit until all the family problems both expressed and detected have been appreciated, and the patient and the relatives have been allowed to bring forth all their anxieties and worries. The Hospice Nurses treat the family circle as well as the patient.

**CONFIDENCE**

Anxiety is one of the shadows which lies over a patient and the family. Often there is a strange reluctance to discuss much of their worries with their own general practitioner. They wish to protect him from any sense of failure which he is bound to feel already and would only be made worse by family pressure. Once the Hospice Nurses have gained their confidence then they are prepared to discuss the situation openly when they realise that they are dealing with a sympathetic person in whom they can trust their peace of mind. The Nurses answer questions truthfully and do not force the issue until the family is prepared for it. No patient should ever be told anything that has to be denied later.

**RELIEF OF PAIN**

About 50% of cases of cancer do not have serious pain, but a small percentage do. A review of causes of admission to St. Christopher's Hospice in Sydenham in London, showed that one of the main factors in requests for admission was pain – the figure was 66.5%. Relief of pain is possible usually by oral drugs which have to be taken regularly and the dose must be titrated accurately. Once the pain has been relieved the patient can live again and join in family activities. In fact, there is often a period of remission in symptoms, and the patient may go home.

**EDUCATION**

The family are supported with technical aids which do not normally exist in the average household, and also by discussions based on the anticipated course of the disease process. Crisis situations should be avoided and both the relatives and patient should be helped to cope with the course of events.

Recognition that not all symptoms in a patient with terminal cancer are due to the malignant condition is considered, so that simple appropriate

*St. Peter's Lodge Tennis Road, Knowle*

remedies may be applied. It is often a great relief to a family circle when the changes are found to be due to something simple which can be alleviated comparatively easily.

Home Care in which the relatives and friends take part helps to reduce the bereavement symptoms. A family that has coped develops an inner feeling of strength and relief. Many husbands, wives or close relatives have not the courage to face a terminal illness, but the nurses give them both practical help and confidence.

Relief of distressing symptoms and anxiety in the home enables visits by friends of the patient to continue to the end phase. Many patients fear loneliness and desertion. The Hospice Service aims to so improve the quality of a patients life both physically and socially that isolation does not occur and the strain of responsibility of the anxious relatives is greatly relieved or even abolished altogether.

St. Peter's Hospice has about 60 patients under treatment at any one time. A waiting list is avoided as far as possible, and early assessment of patients by a visit from the Medical Director is the aim of the unit.

Some patients need not come to St. Peter's at all, and others are so dangerously ill that they do not last long enough to allow the Doctor and nurses to become involved.

The people of Bristol have been wonderful in their support of the Hospice Movement in their own home town. The League of Friends have reached over 2,500 and the volunteers with special skills such as physiotherapy, occupational therapy, hairdressing etc., number about 60. The Nurses can call on 'sitters-in', readers, transport drivers and many others when the necessity arises.

**THE ORGANISATION**

It consists of an Executive Committee with two sub-groups for finance and development. A Council, a Support Committee and House Committee. The only help from the Health Service was £6000 from the Regional Health Authority in the initial phase, but it is to be hoped that the time will come when regular contributions will be made. Patients are admitted without consideration of creed or ability to pay, and the Hospice has depended on voluntary funds both large and small. Trusts as well as individuals have helped, and the week-by-week income just about matches the expenditure. The Support Committee organises many fund-raising activities, but there are many arranged by local people to which St. Peter's personnel are invited. Lectures and talks are given to many groups, and the support which has been generated is a wonderful encouragement to the staff and a great boost to the funds which St. Peter's Hospice requires.

The Hospice Service is a very positive approach to a state from which both the established Services and the general public have tended to shy. The art of medicine is turning the negative into the positive.

**WHAT DOES THE SERVICE OFFER?**

Its aim is to improve the quality of patient's lives both physically and socially; to alleviate isolation and ease the strain of responsibility for anxious relatives by regular visits and support.

**FOR THE FUTURE**

St. Peter's Hospice requires more beds, and there is room on the present site for further building. A Day Centre with occupational therapy for patients under observation might be a very useful help to the Home Care Team and relieve relatives once or twice a week. On the educational side, Courses for Nurses and Doctors and close liaison with the University of Bristol Medical School so that students may be involved in Terminal Care in a practical hospice setting to back up the lectures that they already have in their curriculum. There is a new full-time Medical Director, Dr. Ian Capstick, and supporting staff on the administrative side are being engaged to allow the freedom for patient care and educational activities.

## Figures and Tables

**Figure f1:**
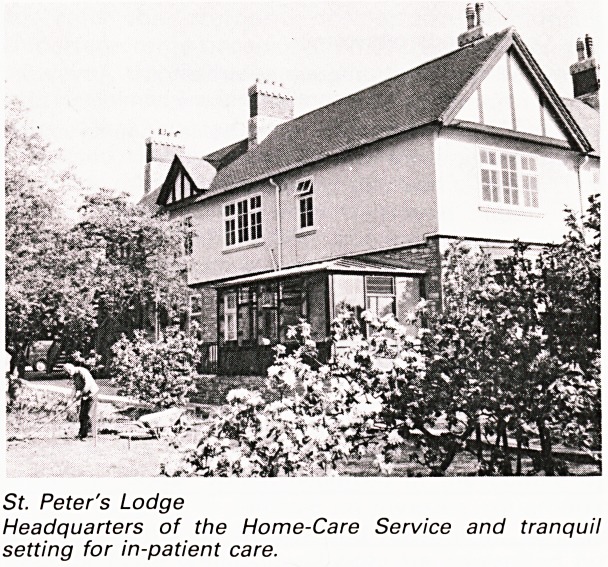


**Figure f2:**